# Predictive Utility of Structured MRI Reporting for Rectal Cancer Outcomes

**DOI:** 10.3390/diagnostics15121472

**Published:** 2025-06-10

**Authors:** Eliodoro Faiella, Filippo Carannante, Federica Vaccarino, Gabriella Teresa Capolupo, Valentina Miacci, Gloria Perillo, Elva Vergantino, Bruno Beomonte Zobel, Marco Caricato, Domiziana Santucci

**Affiliations:** 1 Operative Reasearch Unit of Radiology and Interventional Radiology, Fondazione Policlinico Universitario Campus Bio-Medico di Roma, Via Alvaro del Portillo 200, 00128 Rome, Italy; 2 UOC Chirurgia Colorettale, Fondazione Policlinico Universitario Campus Bio-Medico di Roma, Via Alvaro del Portillo 200, 00128 Rome, Italy

**Keywords:** magnetic resonance imaging, rectal cancer, post-operative complications, local recurrence, structured report, outcome

## Abstract

**Background/Objectives:** This retrospective study evaluates the predictive role of magnetic resonance imaging (MRI) in complications and recurrence in rectal cancer patients undergoing surgery and neoadjuvant therapy, highlighting the impact of structured reporting templates on MRI quality. Compared to traditional free-text reports, structured radiology reports offer a point-by-point evaluation, improving clarity and completeness by thoroughly addressing all relevant findings. MRI is critical in rectal cancer staging, guiding treatment based on tumor characteristics like T stage, sphincter involvement, vascular invasion, and lymph node status. **Methods:** A retrospective analysis of MRI and reports from 67 rectal cancer patients at the time of diagnosis, who were subsequently treated with neoadjuvant radiochemotherapy and surgery, was conducted. MRI report features, including tumor location, morphology, T stage, sphincter infiltration, mesorectal fascia involvement, lymph nodes, and extramural vascular invasion, were evaluated against European Society of Gastrointestinal and Abdominal Radiology (ESGAR) recommendations. Multivariate and univariate analyses were performed to correlate MRI findings with postoperative outcomes such as complications, local recurrence, bleeding, and 30-day anastomotic leaks. **Results:** Sphincter involvement showed a strong association with increased complications (multivariate β = 0.410, univariate r = 0.270). Extramural vascular invasion was linked to higher rates of local recurrence (multivariate β = 0.199, univariate r = 0.127). Lymph node involvement correlated with an elevated risk of postoperative bleeding (multivariate β = 0.133, univariate r = 0.293). Additionally, advanced T staging predicted a higher incidence of 30-day anastomotic leaks (multivariate β = 0.210, univariate r = 0.261). These findings may provide clinically relevant insights to support personalized surgical planning and improve preoperative risk stratification. **Conclusions:** Detailed MRI reporting, aligned with structured templates, significantly guides surgical and therapeutic strategies in rectal cancer management. However, the retrospective nature of the study and the limited sample size may affect the generalizability of the results.

## 1. Introduction

### 1.1. Structured Radiology Report

The benefits of employing structured radiology reports have been widely emphasized in the literature. These advantages, when compared to traditional free-text reports, include enhanced clarity and consistency, greater engagement among radiologists and clinicians, and a more streamlined workflow, making the imaging findings clearer for the multidisciplinary team [1,2]. Furthermore, studies have indicated that standardized reports are more accessible and easier for patients to comprehend [3,4]. Certain types of examinations are particularly suited for structured reporting, especially when they require specific details that are critical for decision-making in patient care. In these cases, using a structured report template can be an effective tool to ensure that all relevant imaging features are included, ensuring that the necessary information is presented clearly and succinctly. MRI for rectal cancer staging is one such example, where numerous objective points must be reported, making structured reporting a valuable approach.

### 1.2. Rectal Cancer MRI Staging

Globally, colorectal cancer stands as the third most common cancer, trailing behind lung and prostate cancer in males and breast and lung cancer in females, with over 1.9 million cases reported in 2022 (1,045,413 in males and 826,706 in females), and it contributed to over 9% of both global cancer incidence and mortality [5,6]. The initial assessment of suspected rectal cancer generally employs a range of diagnostic tools, each offering specific advantages and drawbacks. Commonly used methods include digital rectal examination, endorectal ultrasound, and computed tomography. Digital rectal examination offers useful preliminary insights, such as assessing tumor fixation, sphincter condition, and proximity to the anal verge. However, its role is limited by low sensitivity and specificity, making it unreliable for accurate staging [7]. Endorectal ultrasound is effective for assessing local T staging (particularly T1 and T2) and nodal involvement, especially in distal rectal tumors. However, its accuracy is operator-dependent and limited in evaluating proximal or stenotic lesions [8]. Although computed tomography is valuable for detecting distant metastases essential to staging and treatment planning, its limited soft tissue resolution makes it suboptimal for detailed local assessment of rectal cancer. MRI plays a crucial role in the initial preoperative assessment of rectal cancer, offering high accuracy in tumor staging and identifying imaging features associated with a higher risk of local recurrence [9,10,11]. Rectal cancer patient prognosis is influenced by various factors, such as the extent of tumor invasion within and beyond the bowel wall, the presence of lymph node involvement, extramural vascular invasion, the status of the circumferential resection margin, and the presence of peritoneal tumor involvement [12]. A precise preoperative assessment of these prognostic factors holds significance in the selection of patients for neoadjuvant therapy and in planning surgical strategies aimed at achieving optimal tumor excision, improving patient outcomes, and minimizing complications [13]. A crucial decision influenced by the MRI report provides valuable insights, not only informing the choice of surgical approach but also guiding the selection of neoadjuvant chemoradiotherapy based on tumor and nodal staging [14,15]. There is a strong consensus across major international and national medical societies on the central role of MRI in the diagnostic pathway for rectal cancer, particularly for local staging, the assessment of the circumferential resection margin, and identifying extramural venous invasion. Preferred MRI protocols show substantial alignment across guidelines, with high-resolution T2-weighted sequences serving as the cornerstone for rectal cancer evaluation. Diffusion-weighted imaging is increasingly integrated, particularly for post-treatment restaging. MRI-based TNM classification is generally aligned with the American Joint Committee on Cancer (AJCC) system, though minor variability remains in the interpretation of T and N categories [16]. Training and awareness programs on the appropriate use and interpretation of rectal MRI are essential to ensure diagnostic accuracy and adherence to standardized reporting guidelines.

Neoadjuvant chemoradiotherapy has proven effective in lowering the rates of local recurrence and may improve survival outcomes in specific stages of rectal cancer [17]. However, it is also associated with potential side effects. Consequently, it is essential to accurately identify the patients who are most likely to benefit from this treatment to ensure that it is appropriately targeted. This decision-making process relies heavily on the details provided in the MRI report. The decision-making process is largely driven by the detailed information provided in the MRI report. For clinical decisions to be optimal, the report must comprehensively cover all key findings. Consequently, the use of standardized reporting for rectal cancer staging MRI has been strongly recommended, as outlined in the consensus guidelines issued by the European Society of Gastrointestinal and Abdominal Radiology (ESGAR) [10]. Despite these recommendations, variability persists in the reporting of MRI for rectal cancer staging; indeed, a study even highlighted that only 40% of rectal cancer reports contained the critical information necessary for accurate preoperative staging [12]. Hence, the aim of our study is to assess the impact of implementing a structured report template on the quality of MRI reports for rectal cancer staging, and to evaluate the role of primary staging MRI in predicting complications and/or recurrence in rectal cancer patients who subsequently underwent surgery and radiochemotherapy. To our knowledge, no previous studies in the current literature have simultaneously assessed both these aspects—report quality and prognostic value—within the same patient cohort.

## 2. Materials and Methods

The present study conducted a retrospective analysis of staging MRI exams on a total of 67 patients diagnosed with rectal cancer, who were enrolled between 2011 and 2018, prior to undergoing neoadjuvant radiochemotherapy and subsequent surgical intervention. For each patient, the MRI examination was performed using a 1.5 Tesla scanner (Magnetom Aera, Siemens, Erlangen, Germany) and included T2-weighted, pre-contrast T1-weighted, post-contrast T1-weighted sequences, and diffusion-weighted imaging (DWI). A radiology resident retrospectively reviewed all original MRI reports. The presence or absence of the following key imaging features was assessed: tumor location (low, mid, high), cranio-caudal extension, morphological characteristics, T stage, sphincter involvement, mesorectal fascia involvement, lymph node involvement, and the relationship with the peritoneal reflection. The proportion of reports including each feature was then calculated across the cohort of 67 patients. Patients were excluded if the pre-treatment staging MRI or its corresponding report was not available, or if the MRI examination was incomplete, severely degraded by motion artifacts, or prematurely interrupted due to poor patient compliance.

All these parameters were then correlated with the structured report template suggested by ESGAR guidelines [18].

After staging, all patients underwent neoadjuvant radiochemotherapy and surgical intervention. Patients’ conditions were followed up for at least 5 years. Post-surgical outcomes were reported as present or absent. These included post-surgery local recurrence, postoperative complications (which encompass surgical site infections, bowel obstruction, acute kidney failure, urinary tract infections, as well as pulmonary, cardiovascular, and neurological complications), postoperative bleeding, and 30-day anastomotic leaks.

Then, MRI tumor characteristics were correlated with post-surgical outcomes. The MRI parameters were categorized as follows: cranio-caudal extension (0 if the tumor is less than 3 cm and 1 if greater than 3 cm); morphology (0 for vegetating, 1 for semi-annular, and 2 for circumferential tumors); tumor location (1 for low, 2 for mid, and 3 for the upper rectum); signal restriction in DWI sequences (0 for absent and 1 for present; post-contrast enhancement (0 for absent and 1 for present); T staging (T1, T2, T3, or T4); sphincter involvement (0 for absent and 1 for present); mesorectal fascia involvement (0 for absent and 1 for present); lymph node involvement (0 for absent or 1 for present); extramural vascular invasion (0 for absent and 1 for present). These parameters are summarized in Table 1.

To evaluate the consistency in interpreting MRI features, an inter-rater agreement analysis was conducted among three readers: a second-year radiology resident, a fourth-year radiology resident, and a senior radiologist with seven years of experience. A brief joint training session was conducted prior to image review to ensure consistency in interpretation between readers.

As each regression model included multiple predictors simultaneously, no correction for multiple comparisons was applied within models; moreover, given the exploratory nature of the study, no adjustment was made across the multiple outcome specific.

### Statistical Analysis

Descriptive statistics were used to summarize the clinical data of the patients. All statistical analyses were performed using the SPSS Statistics for Windows version 24.0 software (IBM Corp., Armonk, NY, USA). Pearson correlation coefficient was used to evaluate the relationship between individual MRI parameters and the postoperative outcomes. This statistic ranges from −1 to 1, where 1 indicates a perfect positive linear relationship, −1 indicates a perfect negative linear relationship, and 0 indicates no linear correlation at all. Multiple linear regression analyses were performed to account for any confounding variables.

Regarding the inter-rater analysis, the agreement among the three radiologists was quantified using Cohen’s Kappa coefficient for qualitative features and Bland–Altman plot for quantitative features.

For the multivariate analysis, we employed linear regression models to investigate the relationships between multiple independent MRI parameters and specific postoperative outcomes. Each model was constructed with one of the postoperative outcomes as the dependent variable and MRI characteristics as independent variables. The significance of each regression coefficient was determined using the beta coefficient. The magnitude of the beta coefficients can be interpreted as the strength of the relationship between the predictor variable and the dependent variable. The direction of the beta coefficients (positive or negative) indicates the direction of the relationship between the predictor variable and the dependent variable. A positive beta coefficient means that an increase in the predictor variable is associated with an increase in the dependent variable, while a negative beta coefficient means that an increase in the predictor variable is associated with a decrease in the dependent variable.

## 3. Results

### 3.1. Comparison Between Original Reports and Structured Report

The analysis was conducted based on the content of the original free-text MRI reports. The comparison between the original MRI reports and the structured reporting template revealed notable differences in the frequency of documented features across the 67 cases. The corresponding relative frequencies are summarized in Table 2. Some features were consistently reported, such as lymph node involvement, which was described in all 67 reports (100%), and tumor location, cranio-caudal extension, and morphological characteristics, which were reported in 66 (98.5%) and 63 cases (94.0%), respectively. Conversely, other key features were infrequently included in the original reports. For instance, T staging was mentioned in only 12 cases (18%), and sphincter involvement was documented in just 3 cases (4.5%). Mesorectal fascia involvement was reported in 22 reports (32.8%), while the relationship with the peritoneal reflection was described in only 7 reports (10.4%).

### 3.2. MRI Parameters and Postoperative Outcomes

Among the 67 patients included in the study, 31 experienced postoperative complications (46.3%), 7 developed local recurrence (10.4%), 7 had postoperative bleeding (10.4%), and 13 experienced a 30-day anastomotic leak (19.4%). The correlation between MRI findings and various postoperative outcomes in all patients, based on a retrospective re-evaluation of the MRI images by a radiologist, is shown in Table 3.

- Complications: The most notable finding regarding postoperative complications was the multivariate coefficient for sphincter involvement, with a beta coefficient of 0.410, indicating a strong positive association with increased complications. This was supported by a substantial univariate correlation of 0.270. Despite not reaching traditional levels of statistical significance, the high coefficient underlines the potential risk associated with sphincter involvement in rectal cancer surgeries.
- Local Recurrence: For local recurrence, the extramural vascular invasion stood out with a multivariate coefficient of 0.199, coupled with a univariate correlation of 0.127.
- Postoperative Bleeding: Lymph node involvement was highly correlated with postoperative bleeding, as indicated by both multivariate (0.133) and univariate (0.293) correlation tests. This suggests that the presence of involved lymph nodes could be associated with more complex surgical interventions or inherently aggressive tumor biology, leading to increased risks of bleeding.
- 30-Day Anastomotic Leak: Regarding the 30-day anastomotic leak, T staging presented the highest univariate correlation (0.261), with a notably high multivariate beta coefficient of 0.210, indicating its importance in predicting this severe complication.

The inter-rater agreement analysis demonstrated excellent concordance among the three readers in assessing MRI features. The overall average Cohen’s Kappa coefficient across all pairwise comparisons was 0.89 (95% CI: 0.85–0.93), reflecting high inter-rater reliability. Pairwise agreement was highest between the fourth-year resident and the senior radiologist (K = 0.91), followed by the agreement between the second-year resident and the senior radiologist (K = 0.88). Agreement between the two residents was also strong (K = 0.87). Among the assessed MRI features, the highest agreement was observed for lymph node involvement (K = 0.95) and mesorectal fascia involvement (K = 0.93). Features with slightly lower agreement, while still indicating high concordance, included the extramural vascular invasion (K = 0.82) and sphincter invasion (K = 0.84).

## 4. Discussion

**Figure 1:**
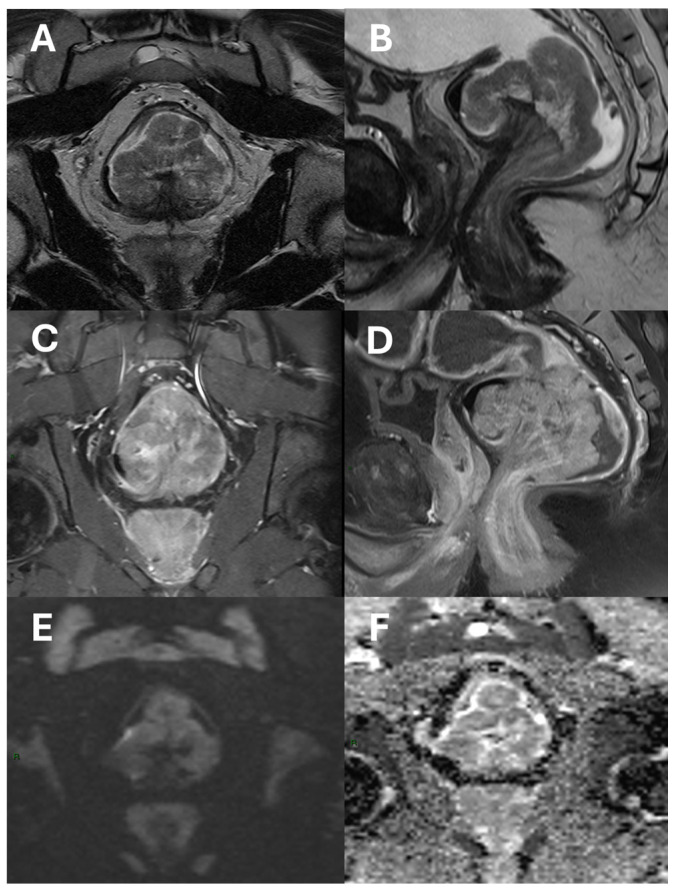
An example of a polypoid adenocarcinoma of the lower rectum raised upon a stalk shown in axial (**A**) and sagittal (**B**) T2-Turbo Spin Echo images; axial (**C**) and sagittal (**D**) T1 Turbo Spin Echo fat-saturated images after contrast-agent administration; diffusion-weighted images (**E**); and ADC map (**F**). The tumor shows post-contrast enhancement and diffusion signal restriction, but it does not extend beyond the mesorectal fat.

Rectal cancer poses a significant global health burden, and the established treatment approach for patients with locally advanced rectal cancer involves neoadjuvant therapy followed by total mesorectal excision [19]. This multimodal treatment has led to a reduction in local recurrence rates; however, it has not significantly improved overall survival outcomes [20,21]. Two recent studies have further highlighted current challenges and evolving strategies in this field. One study demonstrated that a chemoradiotherapy-consolidation chemotherapy protocol can achieve clinical complete response in up to two-thirds of patients, with promising rates of organ preservation in the context of a watch-and-wait approach [22]. Another comparative study reported similar long-term oncologic and functional outcomes between laparoscopic and robotic intersphincteric resections, reinforcing the role of minimally invasive surgery in low rectal cancer [23]. The current prognostic stratification relies on the TNM staging system and tumor regression grading. Nevertheless, TNM’s predictive accuracy is limited by patient variability within the same stage, and tumor regression grading is only available postoperatively, limiting its use in preoperative planning [24]. Therefore, identifying prognostic information from MRI at the time of diagnosis could help optimize treatment and guide patient monitoring from the start. However, to achieve this, a radiology report that is as clear and concise as possible is essential for the multidisciplinary team. Pelvic MRI, indeed, is routinely used as the gold standard for rectal cancer staging, due to its ability to precisely assess possible prognostic indicators, such as extramural vascular invasion and the involvement of the mesorectal fascia [25,26]. High-quality axial MRI scans offer a detailed view of the anatomical structures surrounding the rectum, including muscles, blood vessels, and lymph nodes (Figure 1).

**Figure 2:**
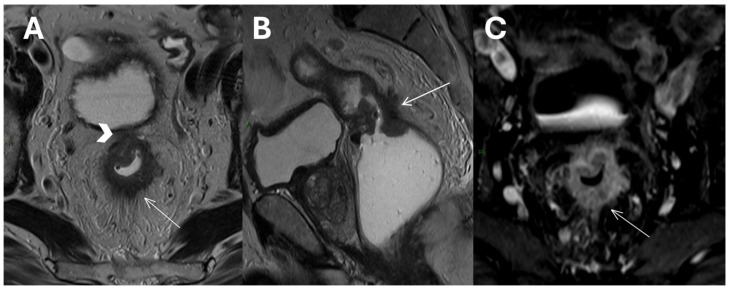
T2-weighted Turbo Spin Echo images in axial (**A**) and sagittal (**B**) planes of a mid-upper rectal tumor showing invasion of the mesorectal fascia (arrowhead), perirectal tumor invasion, and desmoplastic stranding (white arrows). An axial T1 Volumetric Interpolated Breath-hold Examination image after contrast agent administration (**C**).

Additionally, MRI boasts exceptional resolution for soft tissues, enabling highly accurate prediction of both tumor stage (T stage) and mesorectal fascia involvement and effectively assessing the depth of tumor invasion beyond the muscularis propria (Figure 2) [11].

This detailed anatomical information gleaned from high-resolution MRI aids in surgical planning. By meticulously evaluating the extent of tumor infiltration, clinicians can categorize patients based on their individual risk of local and distant recurrence. One major contribution of preoperative MRI in T3 rectal cancer management is its ability to subclassify tumors based on the depth of extramural invasion, revealing prognostic differences within this group [27]. Tumors with limited spread beyond the muscle layer—specifically up to 5 mm—and with no involvement of the circumferential resection margin are generally associated with a more favorable prognosis. In contrast, tumors with deeper extension or with involvement of the surgical margin are linked to a higher risk of recurrence and poorer outcomes. Nonetheless, differentiating between tumors confined to the muscle layer (T2) and those with very minimal extension just beyond it (early T3) remains a diagnostic challenge for magnetic resonance imaging due to the subtle difference in depth [28].

The results of our study highlight the influence of MRI characteristics on the outcomes of rectal cancer surgeries, underscoring the pivotal role that detailed imaging plays in guiding surgical decisions and postoperative care. Overall, these findings strongly support the implementation of structured reporting in MRI assessments. To ensure essential staging elements are accurately included in radiological reports, organizations like ESGAR, Radiological Society of North America (RSNA), Society of Abdominal Radiology (SAR), and various national radiological societies have developed standardized reporting templates, largely based on the TNM staging system proposed by the American Joint Committee on Cancer/Union for International Cancer Control (AJCC/UICC) [10,29,30]. Such reporting ensures comprehensive documentation of all critical aspects, from sphincter and vascular involvement to lymph node and T staging. By doing so, it enhances the accuracy of risk assessments and facilitates the tailoring of surgical and therapeutic strategies to individual patient needs. Our data, in fact, bring to light that despite the high rates of evaluation for tumor location, cranio-caudal extent, and lymph node involvement in original reports, there is a disproportionately low percentage of reports that specify the involvement or non-involvement of the mesorectal fascia, sphincters, and relationship with peritoneal reflections, even though these details are critical for patient management. For example, while tumor location was specified in approximately 98.51% of cases, only about 32.84% of the reports included details on mesorectal fascia involvement, and only 4.48% reported the involvement of the sphincter. This suggests that the structured report template evidently augments the precision of radiological assessments in rectal cancer; it also serves as a valuable tool in ensuring thorough reporting across critical aspects of rectal cancer staging beyond the commonly documented parameters. Several studies in the literature, indeed, have explored how structured reporting enhances the quality and completeness of medical reports [31,32]. For instance, Alvfeldt et al. [33] conducted an illustrative study that evaluated different reporting styles (standardized and structured protocol; standardized semi-structured free-text; and regular free-text). They found that implementing template-based reporting is crucial for adhering to evidence-based practices in reporting rectal cancer using MRI. Their study, in fact, reported completeness rates of 92.9% for the standardized and structured protocol, 77.5% for the standardized semi-structured free-text, and 63.9% for the regular free-text. Despite the advantages of structured reporting, studies show that some interobserver variability still persists. For example, in a recent international multireader study conducted by Najim el Khababi et al. involving 21 radiologists from 12 countries, 75 rectal cancers were staged using an MRI structured reporting template. The results showed high uniformity (interobserver agreement > 80%) for high-risk (≥cT3 ab) and low-risk (≤cT3 cd) features, lateral lymph nodes, tumor deposits, mesorectal fascia, sphincter involvement, and distinguishing between solid and mucinous tumors. However, lower agreement was observed for extramural vascular invasion, detailed cT and cN staging, peritoneal reflection, and tumor morphology [34].

In our study, furthermore, we also assessed the strength and direction of each correlation among MRI parameters to better understand their individual associations with various clinical outcomes.

**Figure 3:**
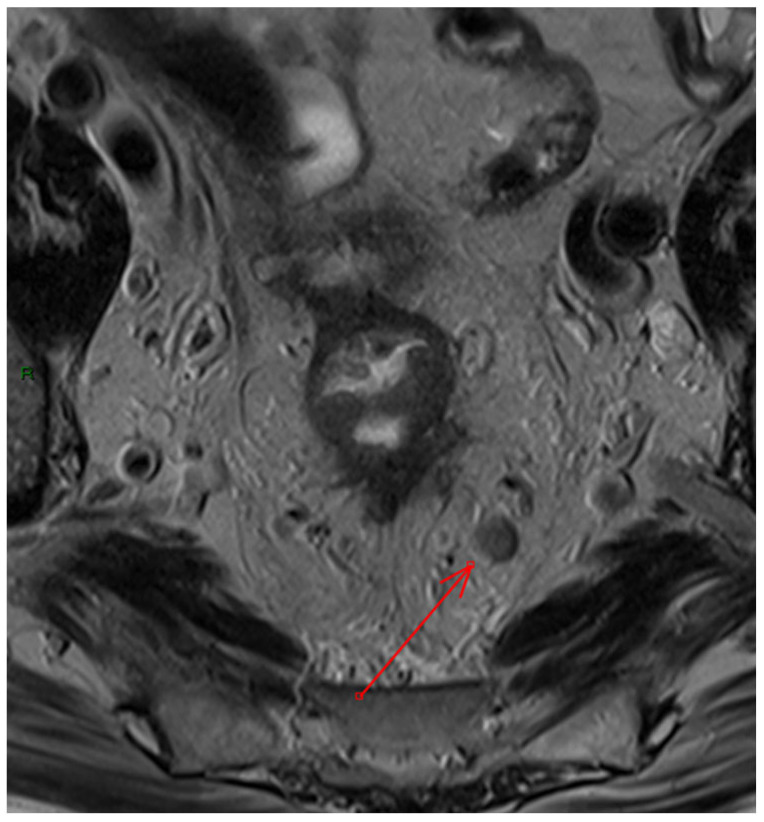
Example of pathological mesorectal lymph node on axial T2 Turbo Spin Echo image (red arrow).

- Complexities of sphincter involvement: Our analysis revealed that sphincter involvement is strongly associated with heightened postoperative complications. This correlation, while not reaching traditional levels of statistical significance, suggests a complexity in surgical approaches when the sphincter is affected. It highlights the need for careful surgical planning and possibly more conservative approaches to preserve sphincter function and reduce complications. The significance of these findings emphasizes the importance of preoperative imaging reviews to better prepare for the challenges that may arise during and after surgery. There remains ambiguity regarding the management of low rectal cancers that affect the anal sphincter complex and the precise delineation, determining their classification as T4b [35]. The Society of Abdominal Radiology’s Colorectal and Anal Cancer Disease-Focused Panel recommends detailed description of anal involvement, specifying the location and length of sphincter muscle affected. This includes identifying involvement of the internal anal sphincter (IAS), intersphincteric space (ISS), or external anal sphincter (EAS) [36]. ESGAR aligns with this detailed approach, advising in detail the involvement of IAS, ISS, and EAS, along with specifying whether the tumor affects the proximal, middle, or lower third of the sphincter complex, and observing any pelvic floor involvement [10]. Providing comprehensive information about sphincter involvement plays a crucial role in the case-by-case decision-making process for patient management, aligning with multidisciplinary team recommendations.
- Extramural vascular invasion as a harbinger of recurrence: The relationship between extramural vascular invasion (EMVI) and local recurrence brings to light the aggressive nature of such tumors. The presence of malignant cells in blood vessels beyond the muscularis propria near a colorectal tumor, indeed, is linked to more advanced tumors and it has been associated with a four-fold increased risk of distant metastases and a significant decrease in disease-free survival, dropping from 74% to 35% [37]. Thus, accurate histological reporting of EMVI is crucial and should be part of structured reporting.
  However, despite this strong association, there is still no consensus on how to tailor treatment strategies based on the positivity of this finding on MRI, and recommendations within the guidelines remain inconsistent [38,39]. EMVI is graded from 0 to 4, with grades 0–2 indicating better outcomes due to the absence of definitive vascular invasion, while grades 3–4 show vascular invasion and are linked to poorer outcomes. Furthermore, EMVI is also associated with higher TNM stage and mesorectal fascia involvement, though few studies have considered other MRI factors like tumor deposits or enlarged lateral lymph nodes, and, to the best of our knowledge, no study has investigated the variations in oncological outcomes between EMVI grades 3 and 4 [40]. The regression of EMVI following neoadjuvant treatment appears to have a positive impact on prognosis [41,42]. However, many studies evaluating EMVI regression are constrained by small sample sizes, which limits their statistical significance. Despite the prognostic significance of EMVI and the need to include it in structured reporting, further research is required to better inform treatment strategies.
- Lymph node involvement and surgical risk: Notably, our study identifies lymph node involvement as a predictive factor for postoperative bleeding. Lymph node involvement is a crucial prognostic factor in rectal cancer, making preoperative neoadjuvant therapy recommended for these patients to help lower the risk of local recurrence [11,43]. Although MRI is the gold standard for staging, it is less accurate for N staging compared to T staging, with sensitivity and specificity ranging from 58 to 77% and 62 to 74%, respectively [44,45,46]. Other imaging methods, such as computed tomography, have demonstrated similar diagnostic accuracy [47]. Our data suggest that the presence of nodal disease, which indicates a more invasive tumor, may require more delicate and extensive surgical interventions, potentially increasing the risk of bleeding. This insight should encourage surgeons to consider preoperative strategies to minimize this risk, such as advanced surgical techniques or preoperative interventions (Figure 3).

- The Critical Role of T Staging in Predicting Anastomotic Leakage: The correlation of higher T stages with the risk of anastomotic leakage (AL) within 30 days post-surgery highlights the importance of accurate T staging. AL is a serious complication in rectal cancer surgery, significantly affecting both short- and long-term outcomes, with rates varying widely from 0% to 36.3% based on anastomosis type and distance from the anal verge. Despite advancements in preoperative assessments and surgical techniques, AL remains a concern, carrying a mortality rate of 2–10% and a 10–100% likelihood of needing a permanent stoma [48,49,50]. AL is classified as “early” or “late” based on diagnosis within or after 30 days post-surgery [51]. AL can be influenced by patient factors (such as malnutrition, immunosuppression, radiation exposure, and obesity [52]), as well as by the type of anastomosis performed (a higher incidence has been observed after end-to-end anastomosis compared to the end-to-side technique [49]), and by tumor size, as highlighted in our study. In fact, a study conducted by Brisinda et al. found that the mean tumor size was larger in patients with AL (47.9 ± 16.1 mm) compared to those without AL (39.0 ± 21.1 mm, *p* = 0.001), correlating this finding with more advanced T stages [53]. These findings emphasize the need for meticulous preoperative planning and possibly adjusting surgical techniques to mitigate this risk.

The high inter-rater agreement observed among the three radiologists, with different years of expertise, highlights the reliability of standardized evaluation of MRI features in rectal cancer staging. In particular, the highest agreement for key parameters such as lymph node involvement and mesorectal fascia status (K = 0.95 and K = 0.93, respectively) demonstrates that rectal MRI can be effectively standardized using a structured reporting template. This finding is particularly encouraging as it suggests that even less experienced radiologists can achieve consistent and reliable assessments when following a systematic approach.

Despite some promising results, it is important to consider several limitations of this study. First, its retrospective design carries an inherent risk of selection bias and limits the ability to draw causal inferences. Additionally, the relatively small sample size may affect the statistical power and generalizability of the findings. The analysis was conducted at a single institution, which may not reflect variability in reporting practices across different centers. Finally, the correlation between MRI features and clinical outcomes could be influenced by confounding factors not fully accounted for. Further prospective, multicenter studies with larger patient cohorts are needed to validate these observations.

## 5. Conclusions

Our study underscores the substantial predictive value of certain MRI parameters—specifically, sphincter involvement, extramural vascular invasion, lymph node involvement, and T staging—for adverse postoperative outcomes. These findings emphasize the critical role of high-quality preoperative MRI in informing surgical planning and anticipating potential complications. Structured reporting emerges as a valuable tool in this context, as it ensures comprehensive assessment of tumor extent and surrounding structures, thereby enhancing the clarity, completeness, and prognostic utility of MRI interpretation. While our results align with existing literature supporting the use of structured templates, the added value of correlating specific imaging features with clinical outcomes reinforces their potential impact on personalized patient management. However, due to the retrospective design and limited sample size, our findings did not reach strong statistical significance. This highlights the need for larger, prospective, multicenter studies to validate these results and further evaluate the clinical impact of structured MRI reporting in rectal cancer management. Moreover, the adoption of standardized structured reporting templates across institutions may enhance consistency and improve the translational value of MRI findings in clinical practice.

## Figures and Tables

**Table 1:** MRI features correlated with post-surgical outcomes.

Cranio-caudal extension	0 = <3 cm 1 = >3 cm
Morphology	0 = Vegetating; 1 = Semi-annular; 2 = Circumferential
Tumor location	1 = Low rectum; 2 = Mid rectum; 3 = Upper rectum
DWI signal restriction	0 = Absent; 1 = Present
Post-contrast enhancement	0 = Absent; 1 = Present
T staging	T1; T2; T3; T4
Mesorectal fascia involvement	0 = Absent; 1 = Present
Lymph node involvement	0 = Absent; 1 = Present
Extramural vascular invasion (EMVI)	0 = Absent; 1 = Present

**Table 2:** The frequency of key features reported in original MRI reports. The column “Number of reports” indicates how many times the variable was explicitly mentioned in the radiology free report, along with the corresponding percentage out of the total number of reports.

Feature	Number of Reports	Percentage (%)
Location (Low, Mid, High)	66	98.51%
Cranio-Caudal Extension	63	94.03%
Morphological Characteristics	63	94.03%
Sphincter Involvement	3	4.48%
T Staging	12	18%
Mesorectal Fascia Involvement	22	32.84%
Lymph Node Involvement	67	100%
Relationship with Peritoneal Reflection	7	10.45%

**Table 3:** The performance of multivariate and univariate analysis was expressed, respectively, by the beta coefficient and P Pearson values. The coefficients vary from −1 to 1. MC: multivariate coefficient; UC: univariate correlation.

	Complications		Local Recurrence/REC		Postoperative Bleeding		30-Day Anastomotic Leak	
MRI Findings	MC	UC	MC	UC	MC	UC	MC	UC
CC extension	0.096	0.150	0.011	0.035	0.153	0.244	−0.179	−0.054
Morphology	−0.140	−0.051	−0.067	0.031	−0.036	0.178	0.027	0.101
Location	0.037	−0.147	0.021	0.048	−0.064	−0.124	−0.033	−0.007
DWI	−0.098	0.020	−0.096	−0.086	−0.027	0.014	−0.018	0.053
CE—T1w	0.132	0.112	0.082	0.087	0.116	0.087	0.095	0.126
T staging	0.065	0.041	0.047	0.072	−0.054	0.001	0.210	0.261
Sphincter involvement	0.410	0.270	−0.148	−0.087	−0.206	−0.087	0.288	−0.126
Mesorectal fascia involvement	0.018	0.172	−0.144	−0.035	−0.033	0.070	−0.169	−0.027
LN involvement	0.065	0.122	0.078	0.142	0.133	0.293	−0.084	0.004
Extramural vascular invasion	0.089	0.095	0.199	0.127	0.036	0.127	0.270	0.084

## Data Availability

The original contributions presented in the study are included in the article, further inquiries can be directed to the corresponding authors.

## Abbreviations

MRI	magnetic resonance imaging
ESGAR	European Society of Gastrointestinal and Abdominal Radiology
SAR	Society of Abdominal Radiology
RSNA	Radiological Society of North America
AJCC	American Joint Committee on Cancer
UICC	Union for International Cancer Control
EAM	external anal margin
EMVI	extramural vascular invasion
IAS	internal anal sphincter
ISS	intersphincteric space
EAS	external anal sphincter
AL	anastomotic leakage

## References

[1] Pesapane F., Tantrige P., Marco P.D., Carriero S., Zugni F., Nicosia L., Bozzini A.C., Rotili A., Latronico A., Abbate F. (2023). Advancements in Standardizing Radiological Reports: A Comprehensive Review. Medicina.

[2] Rocha D.M., Brasil L.M., Lamas J.M., Luz G.V.S., Bacelar S.S. (2020). Evidence of the benefits, advantages and potentialities of the structured radiological report: An integrative review. Artif. Intell. Med..

[3] Park S.B., Kim M.J., Ko Y., Sim J.Y., Kim H.J., Lee K.H., LOCAT Group (2019). Structured Reporting versus Free-Text Reporting for Appendiceal Computed Tomography in Adolescents and Young Adults: Preference Survey of 594 Referring Physicians, Surgeons, and Radiologists from 20 Hospitals. Korean J. Radiol..

[4] Schwartz L.H., Panicek D.M., Berk A.R., Li Y., Hricak H. (2011). Improving communication of diagnostic radiology findings through structured reporting. Radiology.

[5] Shivshankar S., Patil P.S., Deodhar K., Budukh A.M. (2025). Epidemiology of colorectal cancer: A review with special emphasis on India. Indian. J. Gastroenterol..

[6] Cancer Today. https://gco.iarc.who.int/today/.

[7] Krilaviciute A., Becker N., Lakes J., Radtke J.P., Kuczyk M., Peters I., Harke N.N., Debus J., Koerber S.A., Herkommer K. (2023). Digital Rectal Examination Is Not a Useful Screening Test for Prostate Cancer. Eur. Urol. Oncol..

[8] Opara C.O., Khan F.Y., Kabiraj G., Kauser H., Palakeel J.J., Ali M., Chaduvula P., Chhabra S., Lamsal Lamichhane S., Ramesh V. (2022). The Value of Magnetic Resonance Imaging and Endorectal Ultrasound for the Accurate Preoperative T-staging of Rectal Cancer. Cureus.

[9] Gollub M.J., Arya S., Beets-Tan R.G., dePrisco G., Gonen M., Jhaveri K., Kassam Z., Kaur H., Kim D., Knezevic A. (2018). Use of magnetic resonance imaging in rectal cancer patients: Society of Abdominal Radiology (SAR) rectal cancer disease-focused panel (DFP) recommendations 2017. Abdom. Radiol..

[10] Beets-Tan R.G.H., Lambregts D.M.J., Maas M., Bipat S., Barbaro B., Curvo-Semedo L., Fenlon H.M., Gollub M.J., Gourtsoyianni S., Halligan S. (2018). Magnetic resonance imaging for clinical management of rectal cancer: Updated recommendations from the 2016 European Society of Gastrointestinal and Abdominal Radiology (ESGAR) consensus meeting. Eur. Radiol..

[11] Horvat N., Carlos Tavares Rocha C., Clemente Oliveira B., Petkovska I., Gollub M.J. (2019). MRI of Rectal Cancer: Tumor Staging, Imaging Techniques, and Management. RadioGraphics.

[12] Miranda J., Causa Andrieu P., Nincevic J., Gomes De Farias L.D.P., Khasawneh H., Arita Y., Stanietzky N., Fernandes M.C., De Castria T.B., Horvat N. (2023). Advances in MRI-Based Assessment of Rectal Cancer Post-Neoadjuvant Therapy: A Comprehensive Review. JCM.

[13] Dieguez A. (2013). Rectal cancer staging: Focus on the prognostic significance of the findings described by high-resolution magnetic resonance imaging. Cancer Imaging.

[14] Jia X.-X., Wang Y., Cheng J., Yao X., Yin M.-J., Zhou J., Ye Y.-J. (2018). Low- Versus High-Risk Rectal Cancer Based on MRI Features: Outcomes in Patients Treated Without Neoadjuvant Chemoradiotherapy. AJR Am. J. Roentgenol..

[15] Taylor F.G.M., Quirke P., Heald R.J., Moran B., Blomqvist L., Swift I., Sebag-Montefiore D.J., Tekkis P., Brown G., MERCURY study group (2011). Preoperative high-resolution magnetic resonance imaging can identify good prognosis stage I, II, and III rectal cancer best managed by surgery alone: A prospective, multicenter, European study. Ann. Surg..

[16] Awiwi M.O., Jing J.M., Salem U.I., Palmquist S.M., Stanietzky N., Kandemirli V.B., Gjoni E., Surabhi V.R. (2025). Rectal Cancer MRI: An Update. Semin. Roentgenol..

[17] Feeney G., Sehgal R., Sheehan M., Hogan A., Regan M., Joyce M., Kerin M. (2019). Neoadjuvant radiotherapy for rectal cancer management. World J. Gastroenterol..

[18] Granata V., Caruso D., Grassi R., Cappabianca S., Reginelli A., Rizzati R., Masselli G., Golfieri R., Rengo M., Regge D. (2021). Structured Reporting of Rectal Cancer Staging and Restaging: A Consensus Proposal. Cancers.

[19] Bosset J.-F., Collette L., Calais G., Mineur L., Maingon P., Radosevic-Jelic L., Daban A., Bardet E., Beny A., Ollier J.-C. (2006). Chemotherapy with preoperative radiotherapy in rectal cancer. N. Engl. J. Med..

[20] Peeters K.C.M.J., Marijnen C.A.M., Nagtegaal I.D., Kranenbarg E.K., Putter H., Wiggers T., Rutten H., Pahlman L., Glimelius B., Leer J.W. (2007). The TME trial after a median follow-up of 6 years: Increased local control but no survival benefit in irradiated patients with resectable rectal carcinoma. Ann. Surg..

[21] Rödel C., Liersch T., Becker H., Fietkau R., Hohenberger W., Hothorn T., Graeven U., Arnold D., Lang-Welzenbach M., Raab H.-R. (2012). Preoperative chemoradiotherapy and postoperative chemotherapy with fluorouracil and oxaliplatin versus fluorouracil alone in locally advanced rectal cancer: Initial results of the German CAO/ARO/AIO-04 randomised phase 3 trial. Lancet Oncol..

[22] Asoglu O., Bulut A., Aliyev V., Piozzi G.N., Guven K., Bakır B., Goksel S. (2022). Chemoradiation and consolidation chemotherapy for rectal cancer provides a high rate of organ preservation with a very good long-term oncological outcome: A single-center cohort series. World J. Surg. Oncol..

[23] Aliyev V., Piozzi G.N., Bulut A., Guven K., Bakir B., Saglam S., Goksel S., Asoglu O. (2022). Robotic vs. laparoscopic intersphincteric resection for low rectal cancer: A case matched study reporting a median of 7-year long-term oncological and functional outcomes. Updates Surg..

[24] Tumor Regression Grading After Preoperative Chemoradiotherapy for Locally Advanced Rectal Carcinoma Revisited: Updated Results of the CAO/ARO/AIO-94 Trial—Pubmed. https://pubmed.ncbi.nlm.nih.gov/24752056/.

[25] Lord A.C., D’Souza N., Shaw A., Rokan Z., Moran B., Abulafi M., Rasheed S., Chandramohan A., Corr A., Chau I. (2022). MRI-Diagnosed Tumor Deposits and EMVI Status Have Superior Prognostic Accuracy to Current Clinical TNM Staging in Rectal Cancer. Ann. Surg..

[26] Wibe A., Rendedal P.R., Svensson E., Norstein J., Eide T.J., Myrvold H.E., Søreide O. (2002). Prognostic significance of the circumferential resection margin following total mesorectal excision for rectal cancer. BJS Br. J. Surg..

[27] Pratik T., Guo W., Yang C., Bimal R., Zeng M. (2018). Clinical Feasibility Assessment of T3 Sub-Stage in Rectal Cancer Using MRI. IJ Radiol..

[28] Fernandes M.C., Gollub M.J., Brown G. (2022). The importance of MRI for rectal cancer evaluation. Surg. Oncol..

[29] Kassam Z., Lang R., Arya S., Bates D.D.B., Chang K.J., Fraum T.J., Friedman K.A., Golia Pernicka J.S., Gollub M.J., Harisinghani M. (2022). Update to the structured MRI report for primary staging of rectal cancer: Perspective from the SAR Disease Focused Panel on Rectal and Anal Cancer. Abdom. Radiol..

[30] Nougaret S., Rousset P., Gormly K., Lucidarme O., Brunelle S., Milot L., Salut C., Pilleul F., Arrivé L., Hordonneau C. (2022). Structured and shared MRI staging lexicon and report of rectal cancer: A consensus proposal by the French Radiology Group (GRERCAR) and Surgical Group (GRECCAR) for rectal cancer. Diagn. Interv. Imaging.

[31] Al-Sukhni E., Messenger D.E., Charles Victor J., McLeod R.S., Kennedy E.D. (2013). Do MRI reports contain adequate preoperative staging information for end users to make appropriate treatment decisions for rectal cancer?. Ann. Surg. Oncol..

[32] Taylor F., Mangat N., Swift I.R., Brown G. (2010). Proforma-based reporting in rectal cancer. Cancer Imaging.

[33] Alvfeldt G., Aspelin P., Blomqvist L., Sellberg N. (2024). Radiology reporting in rectal cancer using magnetic resonance imaging: Comparison of reporting completeness between different reporting styles and structure. Acta Radiol. Open.

[34] el Khababi N., Beets-Tan R.G.H., Curvo-Semedo L., Tissier R., Nederend J., Lahaye M.J., Maas M., Beets G.L., Lambregts D.M.J. (2023). Pearls and pitfalls of structured staging and reporting of rectal cancer on MRI: An international multireader study. Br. J. Radiol..

[35] Lee M.H., Kim D.H. (2023). Low Rectal Cancers at Initial Staging MRI. RadioGraphics.

[36] Kassam Z., Lang R., Bates D.D.B., Chang K.J., Fraum T.J., Friedman K.A., Golia Pernicka J.S., Gollub M.J., Harisinghani M., Khatri G. (2023). SAR user guide to the rectal MR synoptic report (primary staging). Abdom. Radiol..

[37] Smith N.J., Barbachano Y., Norman A.R., Swift R.I., Abulafi A.M., Brown G. (2008). Prognostic significance of magnetic resonance imaging-detected extramural vascular invasion in rectal cancer. Br. J. Surg..

[38] Glynne-Jones R., Wyrwicz L., Tiret E., Brown G., Rödel C., Cervantes A., Arnold D. (2017). ESMO Guidelines Committee Rectal cancer: ESMO Clinical Practice Guidelines for diagnosis, treatment and follow-up. Ann. Oncol..

[39] Wo J.Y., Anker C.J., Ashman J.B., Bhadkamkar N.A., Bradfield L., Chang D.T., Dorth J., Garcia-Aguilar J., Goff D., Jacqmin D. (2021). Radiation Therapy for Rectal Cancer: Executive Summary of an ASTRO Clinical Practice Guideline. Pract. Radiat. Oncol..

[40] van Geffen E.G.M., Nederend J., Sluckin T.C., Hazen S.-M.J.A., Horsthuis K., Beets-Tan R.G.H., Marijnen C.A.M., Tanis P.J., Kusters M., Aalbers A.G.J. (2024). Prognostic significance of MRI-detected extramural venous invasion according to grade and response to neo-adjuvant treatment in locally advanced rectal cancer A national cohort study after radiologic training and reassessment. Eur. J. Surg. Oncol..

[41] Schaap D.P., Voogt E.L.K., Burger J.W.A., Cnossen J.S., Creemers G.-J.M., van Lijnschoten I., Nieuwenhuijzen G.A.P., Rutten H.J.T., Daniels-Gooszen A.W., Nederend J. (2021). Prognostic Implications of MRI-Detected EMVI and Tumor Deposits and Their Response to Neoadjuvant Therapy in cT3 and cT4 Rectal Cancer. Int. J. Radiat. Oncol. Biol. Phys..

[42] Chand M., Swift R.I., Tekkis P.P., Chau I., Brown G. (2014). Extramural venous invasion is a potential imaging predictive biomarker of neoadjuvant treatment in rectal cancer. Br. J. Cancer.

[43] Distribution of Mesorectal Lymph Nodes in Rectal Cancer: In Vivo MR Imaging Compared with Histopathological Examination. Initial Observations—PubMed. https://pubmed.ncbi.nlm.nih.gov/15868124/.

[44] Zhuang Z., Zhang Y., Wei M., Yang X., Wang Z. (2021). Magnetic Resonance Imaging Evaluation of the Accuracy of Various Lymph Node Staging Criteria in Rectal Cancer: A Systematic Review and Meta-Analysis. Front. Oncol..

[45] Park J.S., Jang Y.-J., Choi G.-S., Park S.Y., Kim H.J., Kang H., Cho S.H. (2014). Accuracy of preoperative MRI in predicting pathology stage in rectal cancers: Node-for-node matched histopathology validation of MRI features. Dis. Colon Rectum.

[46] Al-Sukhni E., Milot L., Fruitman M., Beyene J., Victor J.C., Schmocker S., Brown G., McLeod R., Kennedy E. (2012). Diagnostic accuracy of MRI for assessment of T category, lymph node metastases, and circumferential resection margin involvement in patients with rectal cancer: A systematic review and meta-analysis. Ann. Surg. Oncol..

[47] Li X.-T., Sun Y.-S., Tang L., Cao K., Zhang X.-Y. (2015). Evaluating local lymph node metastasis with magnetic resonance imaging, endoluminal ultrasound and computed tomography in rectal cancer: A meta-analysis. Color. Dis..

[48] Arezzo A., Migliore M., Chiaro P., Arolfo S., Filippini C., Di Cuonzo D., Cirocchi R., Morino M. (2019). REAL Score Collaborators The REAL (REctal Anastomotic Leak) score for prediction of anastomotic leak after rectal cancer surgery. Tech. Coloproctol..

[49] Brisinda G., Vanella S., Cadeddu F., Civello I.M., Brandara F., Nigro C., Mazzeo P., Marniga G., Maria G. (2009). End-to-end versus end-to-side stapled anastomoses after anterior resection for rectal cancer. J. Surg. Oncol..

[50] Daams F., Luyer M., Lange J.F. (2013). Colorectal anastomotic leakage: Aspects of prevention, detection and treatment. World J. Gastroenterol..

[51] Yang S.Y., Han Y.D., Cho M.S., Hur H., Min B.S., Lee K.Y., Kim N.K. (2020). Late anastomotic leakage after anal sphincter saving surgery for rectal cancer: Is it different from early anastomotic leakage?. Int. J. Colorectal Dis..

[52] Sparreboom C.L., van Groningen J.T., Lingsma H.F., Wouters M.W.J.M., Menon A.G., Kleinrensink G.-J., Jeekel J., Lange J.F., Dutch ColoRectal Audit Group (2018). Different Risk Factors for Early and Late Colorectal Anastomotic Leakage in a Nationwide Audit. Dis. Colon Rectum.

[53] Brisinda G., Chiarello M.M., Pepe G., Cariati M., Fico V., Mirco P., Bianchi V. (2022). Anastomotic leakage in rectal cancer surgery: Retrospective analysis of risk factors. World J. Clin. Cases.

